# Prognostic and therapeutic significance of ribonucleotide reductase small subunit M2 in estrogen-negative breast cancers

**DOI:** 10.1186/1471-2407-14-664

**Published:** 2014-09-11

**Authors:** Hang Zhang, Xiyong Liu, Charles D Warden, Yasheng Huang, Sofia Loera, Lijun Xue, Suzhan Zhang, Peiguo Chu, Shu Zheng, Yun Yen

**Affiliations:** Cancer Institute, Second Affiliated Hospital, Zhejiang University School of Medicine, 310009 Hangzhou, Zhejiang China; Department of Molecular Pharmacology, City of Hope National Medical Center and Beckman Research Institute, 1500 E. Duarte Road, 91010 Duarte, CA USA; Bioinformatics Core, Department of Molecular Medicine, City of Hope National Medical Center and Beckman Research Institute, 91010 Duarte, CA USA; Department of Anatomic Pathology, City of Hope National Medical Center and Beckman Research Institute, 91010 Duarte, CA USA

**Keywords:** Ribonucleotide reductase, Breast cancer, ER-negative, Prognostic biomarker

## Abstract

**Background:**

Ribonucleotide reductase (RR) is an essential enzyme involved in DNA synthesis. We hypothesized that RR subunit M2 (*RRM2*) might be a novel prognostic and predictive biomarker for estrogen receptor (ER)-negative breast cancers.

**Methods:**

Individual and pooled survival analyses were conducted on six independent large-scale breast cancer microarray data sets; and findings were validated on a human breast tissue set (ZJU set).

**Results:**

Gene set enrichment analysis revealed that *RRM2*-high breast cancers were significantly enriched for expression of gene sets that increased in proliferation, invasiveness, undifferentiation, embryonic stem/progenitor-like phenotypes, and poor patient survival (p < 0.01). Independent and pooled analyses verified that increased *RRM2* mRNA levels were associated with poor patient outcome in a dose-dependent manner. The prognostic power of *RRM2* mRNA was comparable to multiple gene signatures, and it was superior to TNM stage. In ER-negative breast cancers, *RRM2* showed more prognostic power than that in ER-positive breast cancers. Further analysis indicated that *RRM2* was a more accurate prognostic biomarker for ER-negative breast cancers than the pathoclinical indicators and *uPA*. A new RR inhibitor, COH29, could significantly enhance the chemosensitivity to doxorubicin in ER-negative MDA-MB-231 cells, but not in ER-positive MCF-7 cells.

**Conclusion:**

*RRM2* appears to be a promising prognostic biomarker and therapeutic target for ER-negative breast cancer patients.

**Electronic supplementary material:**

The online version of this article (doi:10.1186/1471-2407-14-664) contains supplementary material, which is available to authorized users.

## Background

Over a million new cases of breast cancer are diagnosed and ~400,000 deaths occur annually [1]. Breast cancer is a heterogeneous disease that has variable gene expression and different outcomes that cannot be predicted by pathologic grade or clinical stages [2, 3]. A comprehensive gene expression signature has identified four major molecular subtypes: luminal A, luminal B, HER2-enriched and basal-like breast cancers, which are largely comprised of the triple negative breast cancer (TNBC) subtype. Each subtype has a distinct clinical behavior and response to therapy [4]. Among the four, the TN and HER2-enriched subtypes are considered to be ER-negative, and account for 15-17% and 15-20% of invasive breast carcinomas respectively [5]. Recently, therapies that specifically target the HER2 receptor have significantly increased the survival of patients with HER2-enriched breast cancers, and PARP (poly-ADP ribose polymerase) inhibition holds some promise as a targeted therapy for TNBC [6, 7]. Gene signatures that include the 70-gene signature [8], 21-gene recurrence score (commercially developed as Oncotype Dx) [9], PI3K signature [10], core serum response signature (CSR) [11], and the grade signature [12], have been developed to predict the survival of breast cancer patients, and have been used to predict the outcome for ER-positive patients. A 7-gene immune response module (IRM) [13] and a HER2-derived prognostic predictor (HDPP, 158 genes) [14] were developed to identify ER-negative breast cancers that were associated with poor prognosis. Multiple-gene-based signatures potentially enhance the accuracy of prediction. However, their disadvantages include higher cost and lack of specific targets for chemotherapeutic agent selection.

Ribonucleotide reductase (RR) is a rate-limiting enzyme essential for DNA replication. RR catalyzes the formation of 2′-deoxyribonucleoside diphosphates from corresponding ribonucleotide diphosphates [15]. Human RR is a heterodimeric tetramer that consists of two large RRM1 subunits and two small subunits, RRM2 and/or RRM2B [16]. *RRM2* overexpression induces cell proliferation and invasion [17, 18] and is positively correlated with higher grade and muscularis propria invasion in bladder [19] and gastric cancers [20]. Previously, we showed that high *RRM2* expression is a prognostic indicator for poor survival for several kinds of cancers, including lung and colorectal cancers [21–23]. RRM2 enhances the invasive capacity of pancreatic adenocarcinoma cells in a NF-kappaB-dependent manner [24], and RRM2 overexpression can increase angiogenesis by down-regulating thrombspondin-1 and increasing production of VEGF [25]. Knocking down *RRM2* also sensitizes cancer cells to the cytotoxic effects of the nucleoside analogs [26]. The *RRM2* gene (mRNA) has been included in some gene signatures, such as the 3D-culture signature [27] and Five-gene Molecular Grade Index (MGI) [28, 29], and the prognostic significance of *RRM2* mRNA has been evaluated [30, 31]. Independent prognostic significance of RRM2 protein needs to be further investigated, and the prognostic performance of *RRM2* (mRNA and protein) also needs to be compared with other existing breast cancer biomarkers or gene signatures. Especially, the prognostic value of RRM2 in ER-negative breast cancers is worth further evaluation.

In this study, we evaluated the prognostic value of *RRM2* by analyzing seven independent breast cancer data sets, both individually and in a pooled manner. *RRM2* expression was significantly correlated with poor survival in a dose-dependent manner, particularly for ER-negative breast cancers. Moreover, the prognostic value of *RRM2* was comparable to the 70-gene signature and 21-gene recurrence score for breast cancer overall, and superior to these biomarkers and pathoclinical indicators for ER-negative breast cancers.

## Methods

### Patients

#### Microarray data sets

A total of 10 published microarray data sets including: Ivshina (GSE4922), Chin (E-TABM-158), Wang (GSE2034), Pawitan (GSE1456), Desmedt (GSE7390), Expo (GSE2109), Huang [32], Bild (GSE3143), Sortiriou (GSE2990) and NKI [33] with clinical annotations were downloaded from the combined microarray dataset BRAVO (Biomarker recognition and validation on-line). The Expo set was excluded because it lacks survival data. Based on BLAST results, the data sets of Huang, Bild and Sortiriou were excluded because of using low similarity probes (36922_at) (Additional file 1: Figure S1*A*). The NKI set was selected for *RRM2* prognostic evaluation because the probe (Agilent Technologies) for RRM2 also is 100% similarity to sequence of > gi|260064012|ref|NM_001165931.1|, and it contains information of most gene signatures’ classification. Finally, six independent microarray data sets include Ivshina [12], Chin [34], Wang [35], Pawitan [36], Desmedt [37] and NKI [33] were chosen for this study. The summarized microarray data sets are shown in Additional file 2: Table S1. The significant correlation between signals yielded from two RRM2 probes (201890_at and 209773_s_at) in the Ivshina set was shown in Additional file 1: Figure S1*B* (R^2^ = 0.736, p < 0.001). Neither 201890_at nor 209773_s_at correlated with the expression of *RRM2B* or *RRM1* (Additional file 1: Figure S1*C to* 1*E*), indicating that the signal was specific for *RRM2* expression.

#### ZJU set

The protocol for the use of human tissues was reviewed and approved by the Institutional Review Board (IRB) of the Second Affiliated Hospital of Zhejiang University (ZJU) (Zhejiang, China). Prior to the study, all patients gave their written informed consent to allow us to use leftover tissue samples for scientific research. All eligible participants had received modified radical mastectomy and the primary tumor samples were obtained from surgical specimens. The exclusion criteria were: 1) no informed consent obtained, 2) multiple cancers, 3) lack of histological diagnosis, and 4) no follow-up information. After applying the selection criteria, a total of 175 breast cancer patients who were diagnosed from 2002 to 2006 were enrolled in the ZJU set (Additional file 2: Table S2). All patients recruited were Chinese females with a median age of 50.0 years (range 20–84 years). The pathoclinical and demographic data was collected by reviewing the hospital records. Of the 175 patients, 46 patients with local advanced breast cancer( Stage IIIA, IIIB and IIIC) received neo-adjuvant chemotherapy before surgery, 140 patients with positive lympho node or aggressive potential(Including: Age <35, Tumor size equal or more than 2 cm, grade 2–3, lymphovascular invasion , and ER/PR negative) were treated with adjuvant chemotherapy (primarily an anthracycline-based regimen) and 9 patients with large tumor size (equal or lager than 5 cm) received post-surgical radiotherapy. After surgery, all patients in the ZJU set were followed up twice a year until September 2010. Median follow-up time was 73.6 months (range: 8.9-104.9) for overall survival (OS) and 71.2 months (range: 2.5-104.9) for progression-free survival (PFS).

### Study design

This is a population-based, retrospective outcome study (Additional file 1: Figure S2). The PFS was defined as the time from initial surgery until tumor recurrence, including local relapse and metastasis. The OS period was calculated as the time from initial surgery to the date the patient was last seen. Only deaths from metastasis and local relapse of breast cancer were considered as the end of the survival period. According to calculations conducted using nQuery Advisor 6.01 software, 170 cases would be sufficient for 80% study power with a two-sided α of 0.05, so the sample size in the ZJU set is sufficient to reach meaningful conclusions.

To normalize the mRNA expression levels among the above data sets, we re-stratified the scores of *RRM2* and other related genes in to four grades (Q1, Q2, Q3 and Q4) based on the percentile for each independently downloaded data set. For Cox analysis, less than the value of the median was regarded as *RRM2*-low, and greater or equal to the median was *RRM2*-high.

### Gene set enrichment analysis (GSEA)

The detailed GSEA protocol can be obtained from the Broad Institute Gene Set Enrichment Analysis website (http://www.broad.mit.edu/gsea) and related references [38]. The GSEA software v2.0.13 was run using a JAVA 7.0 platform. The dataset (.gct) and phenotype label (.cls) files were created and loaded into GSEA software, and gene sets were downloaded from the Board Institute website. The number of permutation was set to 1000, and the phenotype label was *RRM2*-high versus *RRM2*-low. The ranked-list metric was generated by calculating the signal-to-noise ratio, which is based on the difference of means scaled according to the standard deviation.

### Quantitative immunohistochemistry (IHC) assays

The specimen of primary breast cancers specimens in the ZJU set was assembled and built into a Multiple-tissue array (MTA) as previously described [22]. Antibodies against RRM2 and RRM2B were previously generated, selected and tested in our lab [23]. Antibodies against ER (Clone: SP1), PR (Clone: PgR636), HER2 (Clone: A0485) and Ki67 (Clone: MIB-1) were purchased from Dako company, and the p53 antibody (Clone: DO-7) was purchased from Vector Lab. Proteins were stained in tissue sections from the MTAs using a deparaffinization and staining protocol previously described [22]. The cut-off values for ER, PR, HER2 or Ki67 positivity were based on previous reports [2, 39]. Based on the above IHC staining, all participants were classified into four intrinsic subtypes Luminal A, Luminal B, HER2 positive and Basal-like TNBC [40]. For breast cancers, the CD44^+^/CD24^-/low^ was an indicator for the tumor stem/progenitor cells [41]. Double staining of CD44/CD24 was performed using the Autostainer (Dako) and EnVision G|2 Doublestain System for Rabbit/Mouse (DAB+/Permanent Red) (Dako) according to the manufacturer’s instructions. CD44 was detected with diaminobenzidine (DAB) and CD24 was detected with Permanent Red.

RRM2 was located mainly in cytoplasm, but some nuclear staining was also observed. The immunoreactivity in the cytoplasm was scored according to percentage and intensity of the staining. The staining intensity scored as 0: negative, 1: weak, 2: moderate, and 3: strong. Because of heterogeneity of cancer cells, the IHC staining in each cell was varying. In our score system, only more than 10% cells of whole cells with positive staining were taken into consideration. Highest staining staining would be scored final score. Finally, the expression level of the proteins was divided into four subgroups: negative (-), weak positive (+), positive (++), and strong positive (+++)*.* In Cox analysis, (-) and (+) were defined as RRM2-low, whereas (++) and (+++) were defined as RRM2-high.

To avoid observer bias, all the slides were evaluated independently by two different observers with training in pathology in a double-blind manner. Discrepancies were jointly reviewed by the two readers, and missing samples were left blank.

### Small interfering RNA, invasion and cytotoxicity assay

*RRM2* and scrambled control siRNA were purchased from Santa Cruz Biotechnology Inc. Cells were seeded at 2 × 10^5^ cells per well in 6-well plates filled with 2 mL antibiotic-free normal growth medium supplemented with FBS and then incubated at 37°C in a 5% CO_2_ incubator for 24 hours. A total of 4 μL of 10 nmol/mL *RRM2* or control siRNA was transfected into MDA-MB-231 or ZR-75-1 cells by using the transfection RNAiMAX reagent (Invitrogen, Carlsbad, CA). Cells were incubated in the transfection medium for 6 hours and then placed in normal cell culture medium. The inhibition of RRM2 and RRM2B was measured by using Western blot analysis.

For the invasion assay [22], 2.5 × 10^4^ cells were seeded on the Matrigel™ (BD company) insert of the 24-well chamber. After incubation for 72 hours in 5% CO_2_ at 37°C, the top of the Matrigel™ inserts were wiped with a cotton-tipped swab to remove cells that had not migrated through the membrane. The cells on the lower surface of the membrane were stained with 0.5% Coomassie blue (dissolved in 50% ethanol) and counted. Each experiment was performed three times.

Cytotoxicity was assessed using the MTS assay, according to the manufacturer’s instructions (CellTiter 96 AQueous Assay reagent; Promega) on 10 replicates of 2,500 cells per well in a 96-well plate treated with test drugs for 72 hours.

### Data management and statistical methods

The database was downloaded, converted, constructed and managed by using MS-Excel. The JMP 10.0 software (SAS Institution, Cary, NC) was used for general statistical analysis. Group comparisons for continuous data were performed using t-tests for independent means or one-way analyses of variance. Categorical variables were compared using *χ*2 analysis, Fisher’s exact test or the binomial test of proportions. Kaplan-Meier analysis and Cox hazard proportional hazard models were used to analyze OS and PFS. In PFS analysis, patients with distant metastatic breast cancer not completely resected were excluded. Multivariate Cox analysis was applied to adjust for covariate effects, and stratification analysis was used to reduce the confounding effect on estimation of Hazard Ratio (HR). Missing data were coded and excluded from the analysis. Meanwhile the Comprehensive Meta-analysis V2 software was used for meta-analysis. A Fixed-effect model was used to adjusted the HR of *RRM2.*

## Results

### Oncofetal characteristics of *RRM2*in breast cancers

**Figure 1:**
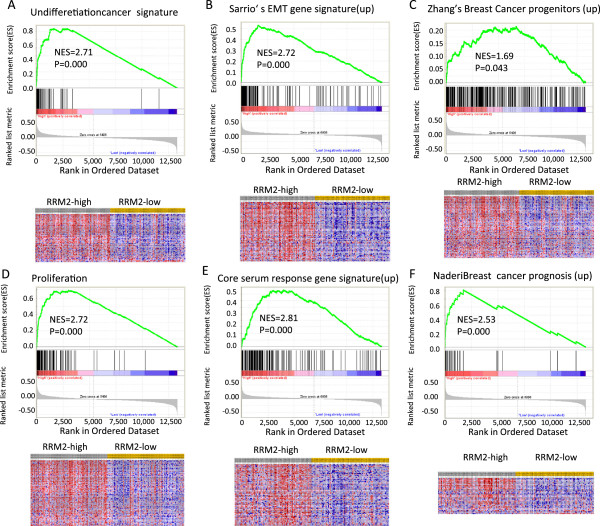
**Enriched gene signatures are associated with aggressiveness in** ***RRM2*** **-high breast cancers.** NES represents the normalized enrichment score for the gene-set enrichment analyses. The ranked list metric was generated by calculating the signal-to-noise ratio, which is based on the difference of means scaled according to the standard deviation. The larger the signal-to-noise ratio, the more distinct the gene expression is for each phenotype, and the more the gene acts as a “class marker.” The Broad Institute Gene Set Enrichment Analysis website (http://www.broad.mit.edu/gsea) provides detailed information about this computational method. The heat maps show the enrichment of genes in the gene sets. Columns are individual samples, and rows represent each gene. Each cell in the matrix represents the expression level of a gene in an individual sample. Red indicates a high level of expression, and green indicates a low level of expression. *RRM2*-high was significantly associated with the following gene sets: **A)** Rhodes undifferentiated cancer gene signature [42], **B)** Sarrio’s epithelial-mesenchymal transition in breast cancer related to basal-like phenotype [43], **C)** Zhang’s Breast cancer progenitor gene signature [44], **D)** Ben-Porath proliferation gene signature [45], **E)**, Chang’s Core Serum Response gene signature [46]**F)** Naderi breast cancer prognosis (up) gene signature [47].

The GSEA was conducted to determine the correlation between *RRM2* expression and oncogenic pathways in the Desmedt set. Samples were re-stratified as *RRM2*-high (equal or greater than median, n = 80) or *RRM2*-low (less than median, n = 79), according to the expression levels of *RRM2* mRNA. Genes that were significantly enriched in *RRM2*-high breast cancer subgroups controlled cancer undifferentiation, Sarrio’s epithelial-mesenchymal transition, breast cancer progenitor-like phenotypes, proliferation gene signature, Chang’s core serum response gene signature, and the Naderi breast cancer prognosis (up) gene signature (Figure 1A through 1 F). These findings suggested that *RRM2* might be associated with poor differentiation and proliferation, and promote the invasiveness of breast cancer.

**Figure 2:**
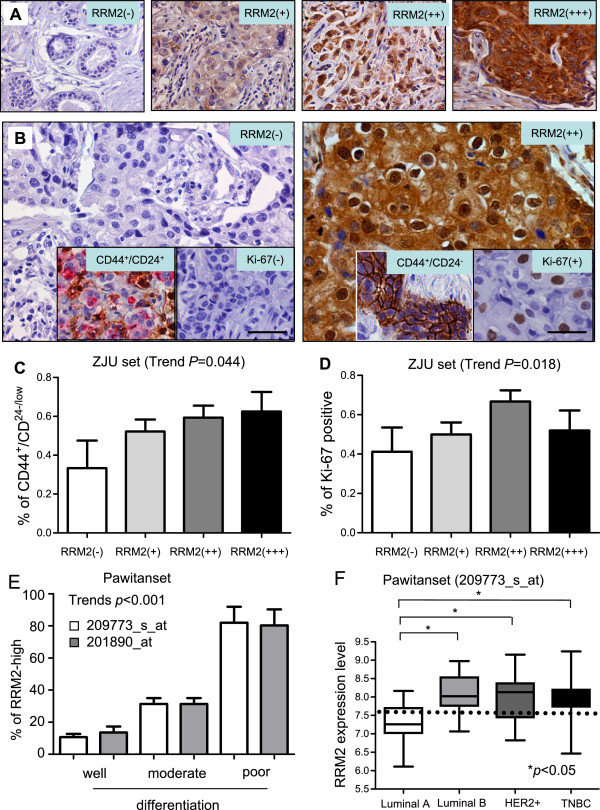
***RRM2*** **is a potential progenitor–like biomarker for breast cancer.** Protein expression levels of RRM2 and other markers were determined by immunohistochemical (IHC) staining. The representative images are: **A)** multiple staining with CD44/CD24, ki-67 and RRM2, **B)** CD44^+^/CD24^+^ sample negative for ki-67 and RRM2 in the left panel, CD44^+^/CD24 ^-/low^ positive for ki-67(+) and RRM2 (+) in right panel. RRM2 is correlated with CD^44+^/CD24^-/low^ and ki-67, as shown in **(C)** and **(D)**, respectively. **(E)** *RRM2* expression was significantly associated with poor differentiation. **(F)** *RRM2* was highly expressed in Luminal B, HER2-positive and TNBC molecular subtypes.

The association of RRM2 protein levels with clinicopathological features of breast cancer was analyzed in the ZJU set. The Figure 2A shows the IHC staining of RRM2-, +, ++ and +++ samples. Increased RRM2 protein levels were associated with larger tumor size, positive lymph nodes and relapse/metastasis (p < 0.05), but not with patient age or histological type (Additional file 2: Table S2). CD44^+^/CD24^-/low^ is considered to be the biomarker for the tumor stem/progenitor cell phenotype in breast cancers [41]. Representative CD44^+^/CD24^+^ and CD44^+^/CD24^-/low^ cells are displayed in Figure 2B. The high expression of RRM2 protein was significantly correlated with CD44^+^/CD24^-/low^, a tumor stem/progenitor cell indicator (Trends *p* = 0.044), and Ki-67, a proliferation indicator (*p* = 0.018) (Additional file 2: Table S2, and Figure 2C and D). In addition, *RRM2* levels were significantly correlated with poorly differentiated breast cancers (*p* < 0.001) (Figure 2E). Moreover, the percentage of RRM2-high in Luminal A, Luminal B/HER2-, Luminal B/HER2+, TNBC and HER2-positive molecular subtypes were 46.0%, 55.9%, 70.0%, 65.4% and 66.7%, respectively (Additional file 2: Table S2). It was indicating that RRM2 protein levels were higher in the molecular subtypes with poor outcomes. The relation between *RRM2* mRNA levels and molecular subtypes of breast cancer also could be seen in the Pawitan set (Figure 2F) (p < 0.05).

### The prognostic significance of *RRM2*for breast cancers

Uni- and multivariate Cox analyses of the data were used to evaluate the prognostic value of *RRM2* for breast cancer patient outcome. The analysis of OS and PFS revealed that high levels of *RRM2* were significantly associated with poor survival in the Desmedt, Pawitan, Wang, Ivshina and NKI sets, except in Chin set. The adjusted HR values for OS were 2.21(95% CI 1.16-4.24), 0.76(95% CI 0.38-1.49), 2.48(95% CI 1.03-6.36) and 2.31(95% CI 1.38-4.02) among the Desmedt, Chin, Pawitan and NKI sets respectively (Table 1), and the adjusted HR values for PFS were 2.16(95% CI 1.32-3.54), 0.80(95% CI 0.40-1.62), 3.28(95% CI 1.43-7.89), 2.20(95% CI 1.44-3.39), 2.21(95% CI 1.37-3.64) and 1.96(95% CI 1.31-2.98) among Desmedt, Chin, Pawitan, Wang, Ivshina and NKI sets respectively. The HR values from the pooled analysis were 2.19 (95%CI 1.28-3.49) and 2.34 (95% CI 1.62-3.41) for OS and PFS respectively. Meta-analysis (Fixed effect model) also yielded similar result (Table 1). It was revealed that *RRM2* mRNA levels were significantly associated with poor outcome in breast cancer patients.

**Table 1:** **Uni- and multivariate analysis for** ***RRM2*** **and survival in microarray data sets**

Data set	Overall survival		Progression-free survival	
	HR (95% CI)	Adjusted HR (95% CI)*	HR (95% CI)	Adjusted HR (95% CI)*
Desmedt set				
	2.27 (1.32-4.04)^‡^	2.21 (1.16-4.24)^‡^	1.75 (1.15-2.68)^‡^	2.16 (1.32-3.54)^‡^
Chin set				
	0.690 (0.37-1.29)	0.76 (0.38-1.49)	0.80 (0.41-1.54)	0.80 (0.40-1.62)
Pawitan set				
	3.10 (1.59-6.49)^‡^	2.48 (1.03-6.36)^†^	3.65 (1.85-7.87)^‡^	3.28 (1.43-7.89)^‡^
Wang set				
	N/A	N/A	2.14 (1.43-3.26)^‡^	2.20 (1.44-3.39)^‡^
Ivshina set				
	N/A	N/A	2.33 (1.45-3.84)^‡^	2.21 (1.37-3.64)^‡^
NKI set				
	3.41 (2.09-5.81)^‡^	2.31 (1.38-4.02)^‡^	2.46 (1.69-3.64)^‡^	1.96 (1.31-2.98)^‡^
Pooled Analysis				
	2.09 (1.26-3.49)^‡^	2.19 (1.28-3.79)^‡^	2.32 (1.72-3.14)^‡^	2.34 (1.62-3.41)^‡^
Meta analysis^#^				
	2.11 (1.57-2.83)^‡^	1.78 (1.28-2.48)^‡^	2.06 (1.70-2.50)^‡^	1.99 (1.62-2.46)^‡^

Note: Uni- and multivariate analysis were conducted to evaluate HR of RRM2 (high vs. low).

*For multivariate analysis, HR was adjusted by age, ER status, Elston Grade in the Desmedt set; In the Chin and Ivshina data sets, HR was adjusted by age. In the Pawitan set, it was adjusted by Elston grade, ER and HER2 status. For the Wang set, HR was adjusted by ER status. The NKI set was adjusted by age and grade.

^#^The meta analysis was conducted unsing Comprehensive Meta-Analysis V2 software with a Fixed –effect model.

^†^Statistical significance, *P* < 0.05; ^‡^Statistical significance, *P* < 0.01.

**Figure 3:**
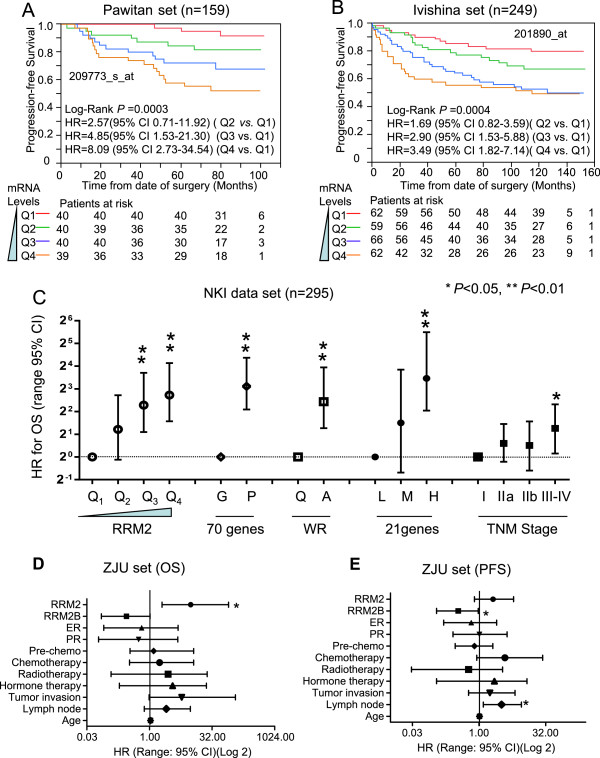
**The prognostic significance of** ***RRM2*** **for the public breast cancer microarray data sets and the ZJU set.** Kaplan-Meier analysis for *RRM2* mRNA levels and PFS of breast cancer patients in the Pawitan set **(A)** and Ivshina set **(B)**. Breast cancer patients were stratified into four subgroups based on their *RRM2* expression levels. Q1 was 0 to the 25th percentile; Q2 was the 25th percentile to the median; Q3 was the median to the 75th percentile; and Q4 was the 75th percentile to the maximum. The subgroups of Q1, Q2, Q3 and Q4 represent mRNA of *RRM2* from low to high. The method used to stratify is described in Materials & Methods. The Cox analyses for *RRM2*, the 70-gene signature, wound-response gene signature, 21-gene recurrence score and TNM stage are shown for the NKI set **(C)**. The protein expression levels of *RRM2* in the ZJU set were determined by IHC and the standard scores are shown in **(D)**. Multivariate Cox analysis for *RRM2* levels and OS are shown in **(E)**. * P < 0.05; **P < 0.01.

*RRM2* was negatively correlated with the survival of breast cancer patients in a dose-dependent manner. Kaplan-Meier analysis revealed that *RRM2* mRNA levels (either 209773_s_at or 201890_at) significantly and negatively impacted the PFS of breast cancer patients in the Pawitan and Ivshina sets (Figure 3A and B). As *RRM2* mRNA levels increased, the outcome for the breast cancer patient became worse, and the relative risk of death increased in a dose-dependent manner (Figure 3C). Similar results were also observed for all others except the Chin set (Additional file 1: Figure S3*A* to *J*).

The prognostic performance of *RRM2* mRNA levels was also compared with some of the prognostic gene signatures and the TNM stage in the NKI dataset. The Cox analyses revealed that the increase in HR was steadily and significantly correlated with increases in *RRM2* mRNA levels (Figure 3C). The prognostic performance of *RRM2* was similar to the 70-genes, wound response genes and 21 gene recurrence score. Also, both the *RRM2* mRNA levels and the gene signatures were more efficient prognostic indicators than the TNM stage.

The prognostic significance of RRM2 protein was also validated in the ZJU set. Multivariate Cox proportional hazard analysis confirmed that high levels of RRM2 protein were significantly and negatively associated with OS of breast cancer patients. After adjusting for RRM2B, ER, PR, pre-operation chemotherapy, adjuvant chemotherapy, radiotherapy and other factors, the HR of RRM2 was 9.84 (95% CI 2.00-80.03, *P* = 0.003) (Figure 3D and E).

### The prognostic significance of *RRM2*in ER-negative breast cancers

**Figure 4:**
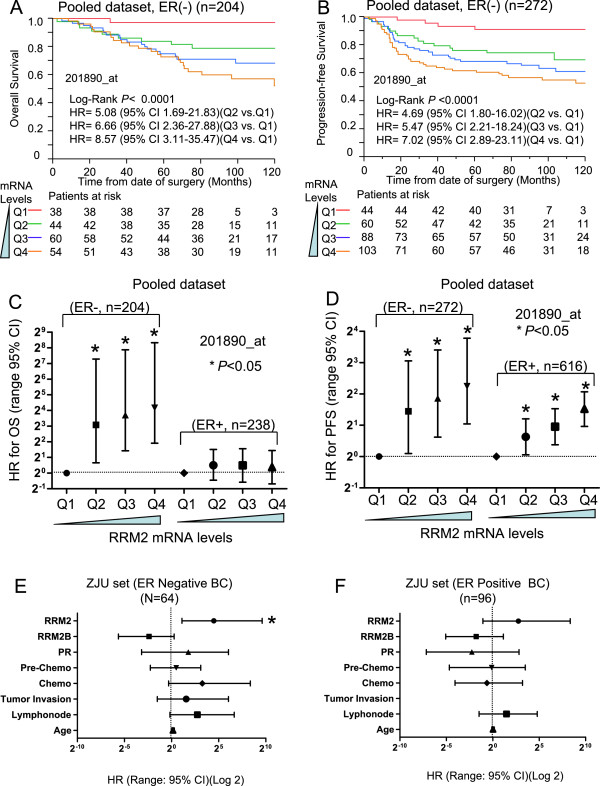
**Prognostic value of** ***RRM2*** **in ER-negative breast cancers.** For the public breast cancer microarray data, we pooled all eligible breast cancers after normalizing. We performed Kaplan-Meier analysis of *RRM2* for the OS **(A)** and PFS **(B)** of breast cancer patients. Cox proportional analysis results are shown for OS and PFS in **(C)** and **(D)**. Findings were verified in the ZJU set. The prognostic performance of RRM2 for ER-negative and ER-positive breast cancers is shown in **(E)** and **(F)**, respectively.

ER-negative breast cancers (including the HER2-positive and TNBC subtypes) have poorer prognosis [48]. A Kaplan-Meier analysis of the Pawitan set revealed that *RRM2* mRNA levels, similar to *uPA,* which is a validated prognostic biomarker for ER-negative breast cancer [49], were significantly associated with poor OS and PFS in a dose-dependent manner in ER-negative, but not ER-positive, breast cancers (Additional file 1: Figure S4). Pooled analysis results also suggested that *RRM2* mRNA levels were significantly correlated with poor OS and PFS (Figure 4A and B). Further multivariate Cox analysis indicated that the HR value for OS increased steadily as the levels of *RRM2* increased in the ER-negative, but not in ER-positive, subgroup (Figure 4C). For PFS, *RRM2* was prognostic for poor survival in both the ER-negative and ER-positive breast cancers, but was more significant for the ER-negative breast cancers (Figure 4D). Similar results were also seen from the ZJU set. A multivariate Cox analysis indicated that the protein levels of RRM2 were significantly associated with poor OS in the ER-negative subgroup (n = 64, HR = 22.4, 95% CI 2.2-285.0) (Figure 4E), but not in the ER-positive subgroup (n = 96) (Figure 4F).

The prognostic value of *RRM2* for the HER2-positive and TNBC subtypes was also evaluated. In the ZJU set, a total of 12 HER2-positive breast cancer and 28 TNBCs were identified. Among these patients, none of the RRM2-low cases (n = 15) died of breast cancer during the following up period (Log rank P = 0.025). However, due to the small number of cases there was no statistical significance in the HER2-positive or TNBC subsets when separated.

### Prognostic performance of *RRM2*in ER-negative breast cancers

**Figure 5:**
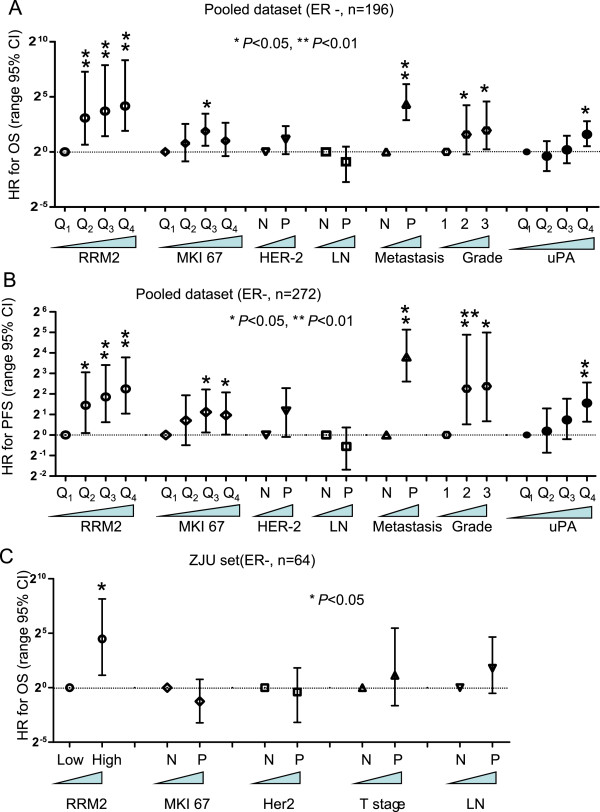
**Validation of the prognostic performance of** ***RRM2*** **for ER-negative breast cancers.** The HR of OS and PFS for various expression levels of *RRM2*, ki-67, HER2, lymphonode involvement, distant metastasis and uPA were determined by Cox proportional analysis. The OS and PFS results are shown in **(A)** and **(B)**. The prognostic performance of RRM2 protein was verified for the ZJU set and is shown in **(C)**.

The efficiency of *RRM2* in predicting the outcome for patients with ER-negative breast cancer was compared with other prognostic markers for this subtype including ki-67, HER2, tumor invasiveness, lymph node involvement, distant organ metastasis, Elson histological stage and uPA. The uPA marker has been reported as a powerful prognostic predictor of breast cancer [49] and its performance was comparable to the Immune Response Module (IRM, 7 genes) and HER2- Derived Prognostic Predictor (HDPP, 158 genes) signatures [14]. In the pooled dataset, the HR of *RRM2* increased steadily in a dose-dependent manner both for OS and PFS (Figure 5A and B). It was comparable to distant organ metastasis for the analysis of both OS and PFS. Furthermore, *RRM2* was more accurate than ki-67, HER2, lymph node involvement, tumor grade and *uPA* for classifying a subgroup of ER-negative breast cancer patients that were at higher risk. Similar results were obtained using the ZJU set (Figure 5C). However, because there are no data about distant metastasis in the ZJU patient set, we could not make this comparison.

### Inhibition of *RRM2*reduced invasion ability and enhanced the drug sensitivity to doxorubicin in ER-negative breast cancer cells

**Figure 6:**
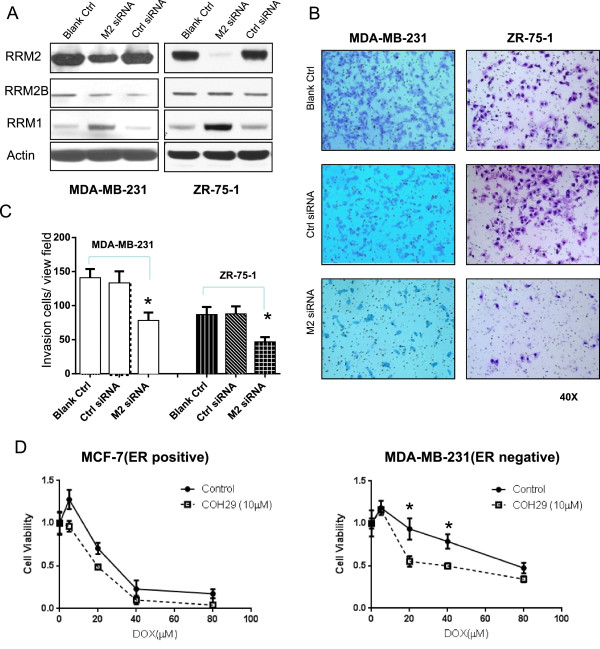
**Specifically inhibiting RRM2 causes reduction of invasion and enhances the drug sensitivity to doxorubicin in ER negative breast cancer cells. (A)** Western blot analysis showed a decrease in RRM2 caused by siRNA in MDA-MB-231 and ZR-75-1 cells. **(B)** Decrease of cancer cells invasive ability by *RRM2* siRNA. **(C)** Summary of cancer cell invasion assay. * p < 0.05 in compared with control siRNA (Ctrl siRNA). **(D)** Assays of cytotoxicity to doxorubicin (DOX) were conducted on MCF-7(ER positive) and MDA-MB-231 (ER negative) cell lines. Both cell lines were seeded and pretreated with or without 10 μM of COH29 for 24 hours, and then cells were treated with different dose of DOX. Each point on the survival curve was normalized to the corresponding value of the initiating point. The sensitivity to doxorubicin was significantly enhanced by the RR inhibitor, COH29, in MDA-MB-231 cells (*p < 0.05), but not in MCF-7 cells.

The above findings suggested that RRM2 inhibitors might be a promising anti-cancer agent for reducing invasion ability and enhancing the chemosensitivity in ER-negative breast cancers. Here, the *RRM2* siRNA was used to inhibit the expression of RRM2 in MDA-MB-231(ER-negative) and ZR-75-1(ER-positive) breast cancer cell lines. It was shown that the protein expression levels of RRM2 were significantly reduced by *RRM2* siRNA in both cell lines (Figure 6A). As with inhibition of RRM2, the invasion abilities of cells were significantly reduced about 50% in both MDA-MB-231 and ZR-75-1 cells (p < 0.05) (Figure 6B and C). This phenomenon also could be seen in MCF-7, another ER-positive breast cancer cell line (Data not shown). This is compatible with our previous findings [23, 50].

A novel RR inhibitor, COH 29, was reported to inhibit RR activity by blocking RRM2 binding to RRM1, thus inactivating the RR holoenzyme [51]. Here, we have preliminarily demonstrated that inhibition of RRM2 by COH29 could significantly reduce the growth of MCF-7 and MDA-MB-231 breast cancer cells. Also, we found that COH29 also could significantly enhance the chemosensitivity of breast cancer cells to doxorubicin in ER-negative MDA-MB-231 cells, but not in ER-positive MCF-7 cells (Figure 6D). This synergetic effect also could not be seen in ZR-75-1 cells (Data not shown). Therefore, the RRM2 might be served as a therapeutic biomarker for application of RR inhibitor to treatment of ER-negative breast cancers.

## Discussion

A recent investigation demonstrated that the reproducibility of current preclinical cancer researches was only 11% [52]. A similar result was also seen in another report [53]. Therefore, third party validations play a very important role in proving the reliability of research results. Open access public microarray databases provided an opportunity for researchers to validate their findings in a non-biased platform. Here, we demonstrated that *RRM2* impacted the survivability of breast cancer patients in 6 of 7 independently microarray data sets (except Chin’s set), which indicates the high reproducibility of this result. The reason why RRM2 did not impact poor survival in Chin’s set might be of the small sample size (only 118 cases), which did not have enough power to detect significant differences. We showed that increased *RRM2* expression was accompanied by increased expression of other widely used biomarkers and gene sets of proliferation, undifferentiation, and stem/progenitor phenotypes. The role of *RRM2* in malignancy has been demonstrated in several solid cancers including bladder [19], pancreatic [54], gastric [20] and colorectal cancers [23], and has been attributed to increased proliferation and invasion [20]. Previously, gene expression profiling has uncovered molecular signatures that influence the choice for breast cancer treatment. Four distinct subgroups are characterized by different gene expression patterns and show diverse epidemiologic, histopathology and clinical features, respond to different therapeutic strategies. Multiple studies have shown the outcomes are much poorer for patients with ER-negative breast cancer [8]. This study demonstrated that either protein or mRNA of *RRM2* was highly expressed in the molecular subtypes of TNBC, HER-2 positive and Luminal B, which were subtypes with relative poorer outcome. The protein expression of *RRM2* was also positively and significantly associated with CD44^+^/CD24^-/low^, a tumor stem/progenitor cell biomarker. In addition, *RRM2* levels were significantly correlated with poorly differentiated breast cancers (*p* < 0.001). Overall, *RRM2* was significantly related to aggressiveness and associated with poor outcome in breast cancers, especially in ER-negative breast cancer. These findings were validated in many independent studies including participants with different genetic and socio-economic background, which indicates the reliability of this study. In 3D–signature study, the RRM2 could not impact poor survivability in ER negative breast cancer [27]. This is because there were insufficient ER negative samples in their study.

Currently, prognostic/predictive factors including primary tumor size, lymph node stage, histological grade and hormone receptor status are used routinely in clinical practice to choose systemic therapies [55]. However, breast cancers are significantly heterogeneous even between patients whose tumors have similar clinical and pathological characteristics. Therefore, distinguishing patients who will be more prone to relapse and will need more intensive therapy is an urgent problem for oncologists. Based on high throughput gene expression, a series of multiple gene signatures have been developed for predicting the survival of breast cancer patients. *RRM2* also has been included in some gene signatures [27–30]. Overall, our data also indicate that the 70-gene, 21-gene recurrence score and wound-response gene signatures are more efficient than TNM stage for predicting patient outcome. Although multiple-gene-based signatures significantly enhance the accuracy of prediction, they also dramatically increase the diagnostic cost. Interestingly, the prognostic performance of *RRM2* was comparable to all of the above gene signatures for breast cancers overall in the NKI set. Furthermore, *RRM2* was more accurate for predicting the outcome of patients with ER-negative breast cancer than uPA, Ki-67, HER2 and Elson grade. Therefore, determining the *RRM2* mRNA or protein levels could be more accurate and cost-effective for predicting the outcome of patients with ER-negative breast cancers.

Out of all breast cancers diagnosed, approximately 15% to 20% are HER2-enriched and another 15%-17% are TNBC [48]. These two types typically experience a poorer prognosis than the ER-positive subtypes and biomarkers predicting their prognosis and therapeutic response are urgently needed. Several studies have revealed that RRM2 expression is also related to resistance to gamma radiation and chemotherapeutic agents [56, 57], which is presumably due to the essential role of RRM2 in DNA repair [58]. Many studies have shown that using small interfering RNA (siRNA) to inhibit *RRM2* overexpression significantly reverses cancer cell resistance to chemotherapy agents and gamma radiation [56, 57]. In a previous study, we demonstrated that using hydroxyurea to inhibit RRM2 significantly enhanced the chemosensitivity of KB cells (a head and neck cancer cell line) to gemcitabine [59]. Cell culture studies also indicate that expression of siRNAs targeting RRM2 significantly reduced the invasion ability in MDA-MB-231 and ZR-75-1 breast cancer cells, which was consistent with our previous studies on other cancers [23, 50]. Similar to the way that HER2 is used to identify subtypes that are likely to respond to trastuzumab, *RRM2* may be a predictive marker that can stratify the more fatal types of ER-negative breast cancers to identify breast cancer subtypes that will respond to RRM2 inhibitors. Alternatively, sorting patients who have ER-negative breast cancers according to their levels of *RRM2* expression may prevent over-treatment of the low expressers. The COH29 is a newly developed RR inhibitor that inhibits RR activity by blocking RRM2 binding to RRM1, thus inactivating the RR holoenzyme [51]. Here, our pilot study indicated that inhibition of RRM2 by COH29 could significantly reduce the growth of breast cancer cells. Also, it could significantly enhance the sensitivity of MDA-MB-231 (ER-negative) cell to doxorubicin, a common therapeutic agent for breast cancers. Nevertheless, we will perform further clinical trials using novel *RRM2* inhibitors (such as *RRM2* siRNA nanoparticles and COH29) to address this hypothesis. According to our data, further investigating and understanding the effect of *RRM2* expression on breast cancer malignancy could lead to a novel approach for treating ER-negative breast cancers.

## Conclusions

Our study revealed *RRM2* was not only an indicator for breast cancers’ proliferation and invasiveness, but also a biomarker for undifferentiation and stem/progenitor-like phenotypes. The prognostic performance of *RRM2* was superior to the current pathology stages and biomarkers in ER-negative breast cancers. Targeting on *RRM2* might be a promising therapeutic method to enhance the chemosensitivity for ER-negative breast cancer patients.

## Electronic supplementary material

Additional file 1: Figure S1: Validate the quality of *RRM2* Probes. **Figure S2.** Study design of outcome study. **Figure S3.** Kaplan-Meier analysis for *RRM2* and outcome of BCs among downloaded published data sets. **Figure S4.** Prognostic performance of *RRM2* and uPA in ER negative BC. (PPT 346 KB)

Additional file 2: Table S1: Overall review of Published microarray data sets. **Table S2.** Demographic characteristics and distribution of RRM2 high in ZJU set. (DOC 94 KB)

## Abbreviations

RR: Ribonucleotide reductase
RRM1: Human ribonucleotide reductase large subunit M1
RRM2: Human ribonucleotide reductase small subunit M2
RRM2B: a p53 dependent human ribonucleotide reductase small subunit R2 (p53R2)
ER: Estrogen receptor
TNBC: Triple negative breast cancer
FFPE: Formalin-fixed, paraffin-embedded tissue block
PARP: Poly ADP ribose polymerase
IHC: immunohistochemistry
siRNA: short interfering RNA
MTS: (3-(4,5-dimethylthiazol-2-yl)-5-(3-carboxymethoxyphenyl)-2-(4-sulfophenyl)-2H-tetrazolium)
dNTP: Deoxyribonucleoside triphosphate
NDP: Ribonucleoside diphosphate
GSEA: Gene set enrichment analysis
OS: Overall survival
PFS: Progression- free survival
OR: Odds ratio
HR: Proportional hazard ratio
95% CI: 95% confidence interval
IRM: Immune response rmodule
HDPP: HER2- derived prognostic predictor.
