# Supplementary material for: Prognostic and therapeutic significance of ribonucleotide reductase small subunit M2 in estrogen-negative breast cancers
Source: BMC Cancer. 2014 Sep 11;14:664. doi: 10.1186/1471-2407-14-664 (PMC4171582; doi:10.1186/1471-2407-14-664)
Supplement: Supplementary file 1 — Additional file 1: Figure S1: Validate the quality of RRM2 Probes. Figure S2. Study design of outcome study. Figure S3. Kaplan-Meier analysis for RRM2 and outcome of BCs among downloaded published data sets. Figure S4. Prognostic performance of RRM2 and uPA in ER negative BC. (PPT 346 KB) [file 12885_2014_4849_MOESM1_ESM.ppt]

## Slide 1
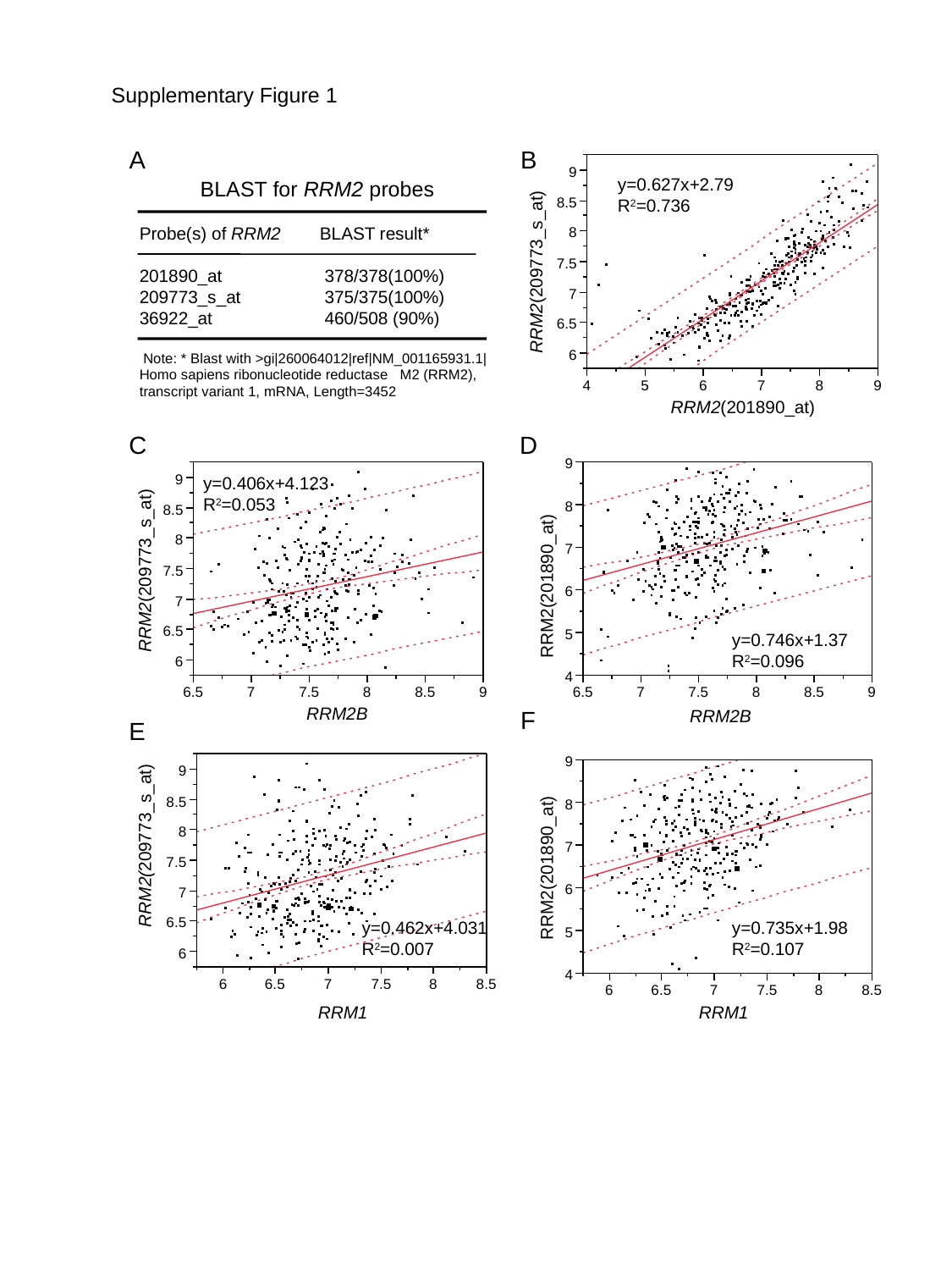

# Supplementary Figure 1
A
B
y=0.627x+2.79
R2=0.736
BLAST for RRM2 probes
Probe(s) of RRM2 BLAST result*
201890_at	 378/378(100%)
209773_s_at	 375/375(100%)
36922_at	 460/508 (90%)
 Note: * Blast with >gi|260064012|ref|NM_001165931.1| Homo sapiens ribonucleotide reductase M2 (RRM2), transcript variant 1, mRNA, Length=3452
RRM2(209773_s_at)
RRM2(201890_at)
C
D
y=0.406x+4.123
R2=0.053
RRM2(209773_s_at)
RRM2(201890_at)
y=0.746x+1.37
R2=0.096
RRM2B
F
RRM2B
E
RRM2(209773_s_at)
RRM2(201890_at)
y=0.462x+4.031
R2=0.007
y=0.735x+1.98
R2=0.107
RRM1
RRM1

## Slide 2
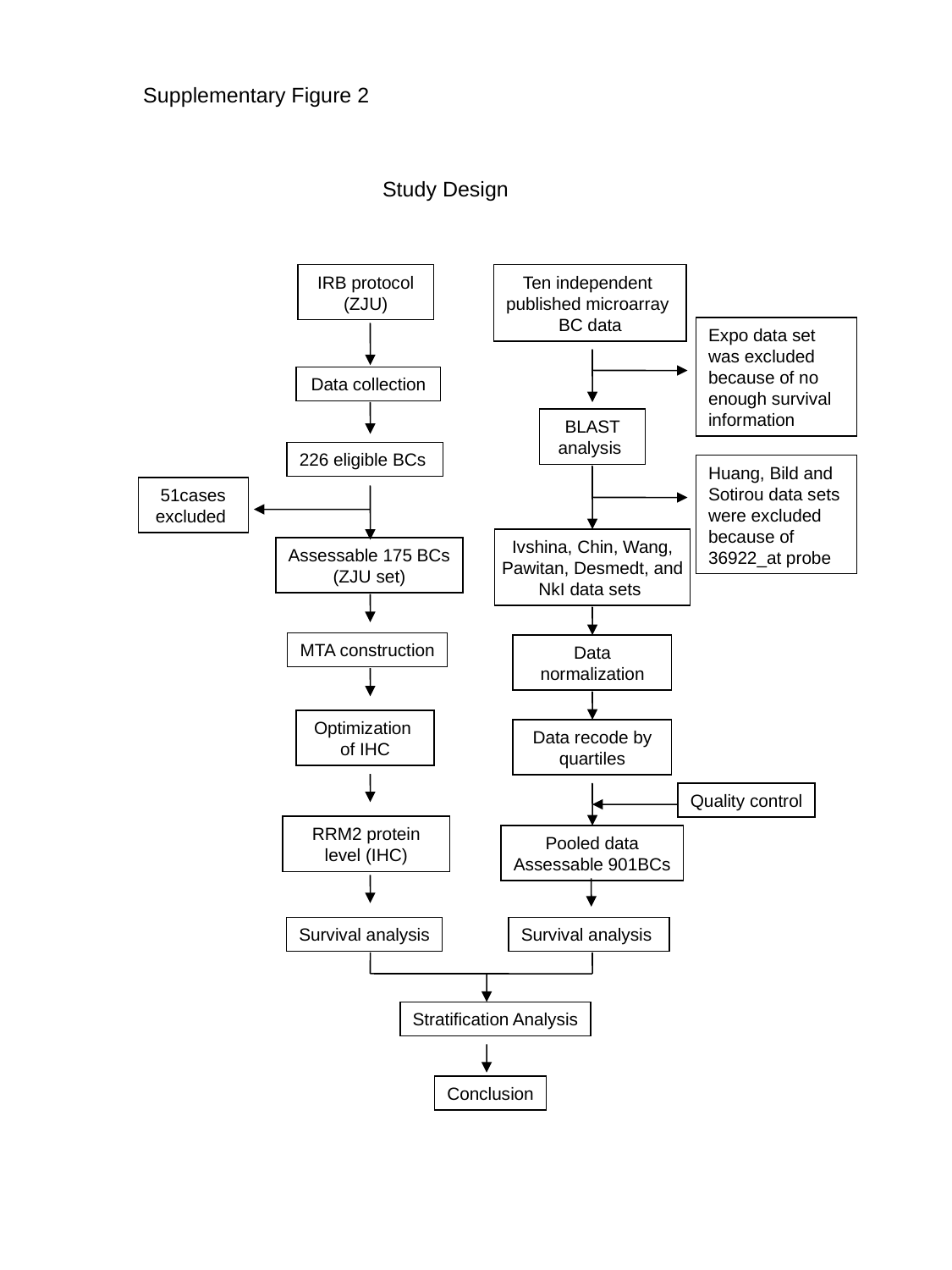

Supplementary Figure 2
Study Design
IRB protocol
(ZJU)
Ten independent
published microarray
BC data
Expo data set was excluded because of no enough survival information
Data collection
BLAST analysis
226 eligible BCs
Huang, Bild and Sotirou data sets were excluded because of 36922_at probe
51cases
 excluded
Ivshina, Chin, Wang, Pawitan, Desmedt, and NkI data sets
Assessable 175 BCs
(ZJU set)
MTA construction
Data normalization
Optimization
of IHC
Data recode by quartiles
Quality control
RRM2 protein level (IHC)
Pooled data
Assessable 901BCs
Survival analysis
Survival analysis
Stratification Analysis
Conclusion

## Slide 3
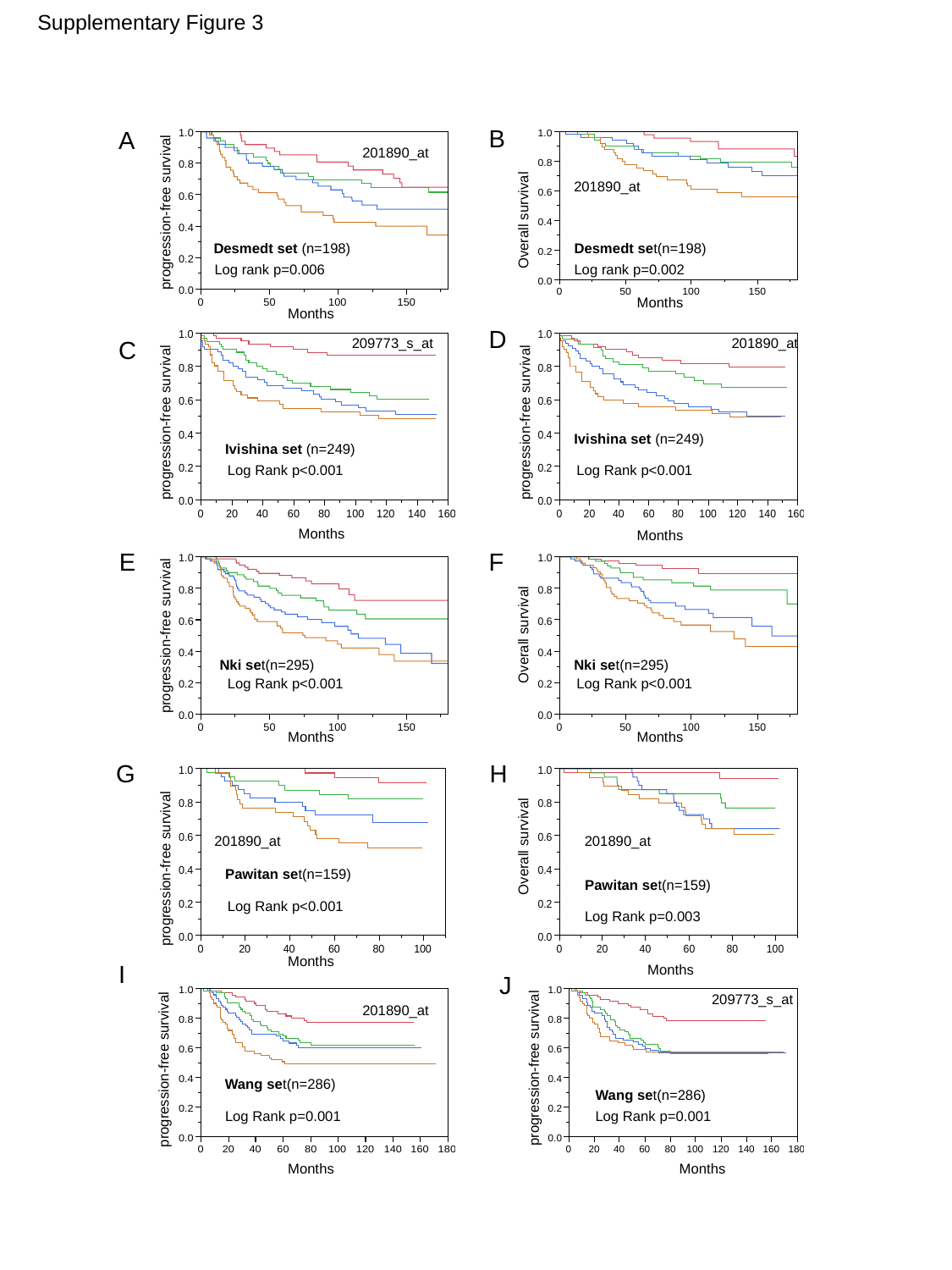

Supplementary Figure 3
B
A
201890_at
201890_at
progression-free survival
Overall survival
Desmedt set (n=198)
Desmedt set(n=198)
Log rank p=0.006
Log rank p=0.002
Months
Months
D
C
209773_s_at
201890_at
progression-free survival
progression-free survival
Ivishina set (n=249)
Ivishina set (n=249)
Log Rank p<0.001
Log Rank p<0.001
Months
Months
E
F
Overall survival
progression-free survival
Nki set(n=295)
Nki set(n=295)
Log Rank p<0.001
Log Rank p<0.001
Months
Months
G
H
201890_at
201890_at
Overall survival
progression-free survival
Pawitan set(n=159)
Pawitan set(n=159)
Log Rank p<0.001
Log Rank p=0.003
Months
I
Months
J
209773_s_at
201890_at
progression-free survival
progression-free survival
Wang set(n=286)
Wang set(n=286)
Log Rank p=0.001
Log Rank p=0.001
Months
Months

## Slide 4
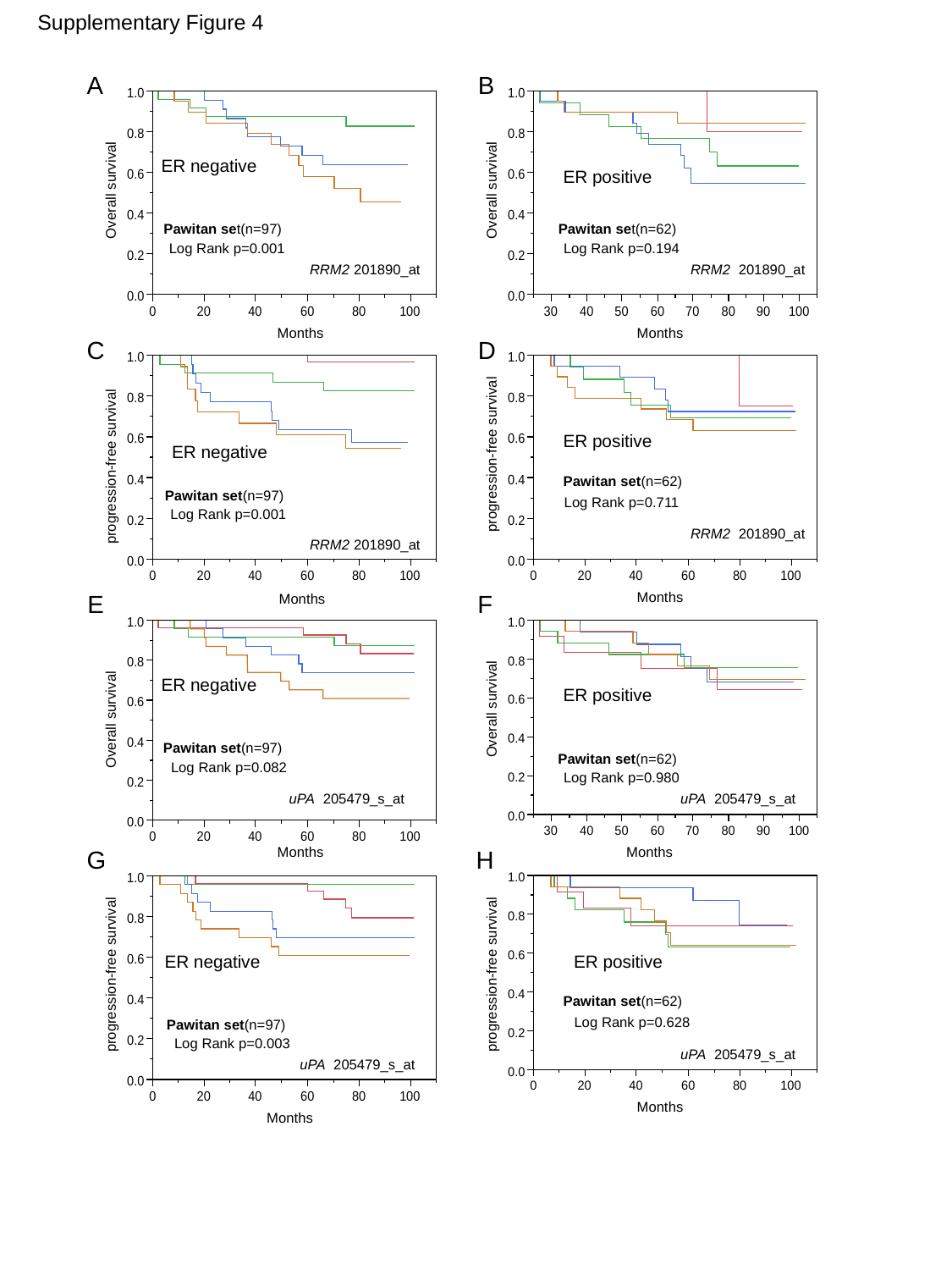

Supplementary Figure 4
A
B
ER negative
ER positive
Overall survival
Overall survival
Pawitan set(n=97)
Pawitan set(n=62)
Log Rank p=0.001
Log Rank p=0.194
RRM2 201890_at
RRM2 201890_at
Months
Months
C
D
ER positive
ER negative
progression-free survival
progression-free survival
Pawitan set(n=62)
Pawitan set(n=97)
Log Rank p=0.711
Log Rank p=0.001
RRM2 201890_at
RRM2 201890_at
F
Months
E
Months
ER negative
ER positive
Overall survival
Overall survival
Pawitan set(n=97)
Pawitan set(n=62)
Log Rank p=0.082
Log Rank p=0.980
uPA 205479_s_at
uPA 205479_s_at
Months
Months
G
H
ER negative
ER positive
progression-free survival
progression-free survival
Pawitan set(n=62)
Log Rank p=0.628
Pawitan set(n=97)
Log Rank p=0.003
uPA 205479_s_at
uPA 205479_s_at
Months
Months
