# Supplementary material for: Prognostic and therapeutic significance of ribonucleotide reductase small subunit M2 in estrogen-negative breast cancers
Source: BMC Cancer. 2014 Sep 11;14:664. doi: 10.1186/1471-2407-14-664 (PMC4171582; doi:10.1186/1471-2407-14-664)
Supplement: Supplementary file 2 — Additional file 2: Table S1: Overall review of Published microarray data sets. Table S2. Demographic characteristics and distribution of RRM2 high in ZJU set. (DOC 94 KB) [file 12885_2014_4849_MOESM2_ESM.doc]

**Supplementary Table 1: Overall review of published microarray data sets**

| **Data set** | **Ivshina** | **Chin** | **Wang** | **Pawitan** | **Desmedt** | **NKI^#^** |
| --- | --- | --- | --- | --- | --- | --- |
| No. of patients | 249 | 130 | 286 | 159 | 198 | 295 |
| Assessable cases* | 213 | 118 | 253 | 159 | 158 | 295 |
| Date of study | 1987-1989 | 1989-1997 | 1980-1995 | 1994-1996 | 1980-1998 | 1984-1995 |
| Microarray | AffimetrixHG-U133 | AffimetrixHG-U133 | AffimetrixHG-U133 | AffimetrixHG-U133 | AffimetrixHG-U133 | Agilent  25K Chip |
| Accession No. | GSE4922 | [Experiment E-TABM-158](http://www.ebi.ac.uk/arrayexpress/experiments/E-TABM-158) | GSE2034 | GSE1456 | GSE7390 | N/A |
| *RRM2* probes |  |  |  |  |  |  |
| 20189_at | Y | Y | Y | Y | Y | N/A |
| 209773_s_at | Y | Y | Y | Y | Y | N/A |
| Age at diagnosis | 63  (28-93) | 51  (31-88) | N/A | N/A | 46  (24-60) | 44  (26-53) |
| Hist. type | N/A | N/A | N/A | N/A | Y | Y |
| Elson grade | Y | Y | N/A | Y | Y | Y |
| Tumor size | Y | Y | N/A | N/A | Y | Y |
| Lymph node | Y | Y | Y | N/A | Y | Y |
| Metastasis | N/A | N/A | Y | N/A | Y | Y |
| AJCC stage | Y | Y | N/A | N/A | Y | Y |
| ER status | Y | Y | Y | Y | Y | Y |
| PR status | N/A | Y | N/A | N/A | N/A | Y |
| HER2 status | N/A | Y | N/A | Y | N/A | Y |
| MKI67 status | N/A | N/A | N/A | N/A | N/A | Y |
| P53 mutation | Y | Y | N/A | N/A | N/A | N |
| Molecular subtype | N/A | N/A | N/A | Y | N/A | Y |
| Chemotherapy | N/A | Y | N/A | N/A | N/A | Y |
| Radiotherapy | N/A | Y | N/A | N/A | N/A | Y |
| Hormone therapy | N/A | N/A | N/A | N/A | N/A | Y |
| OS months†  (Range) | N/A | 1.6-170.4 | N/A | 2.2-101.9 | 4.9-303.6 | 1.0-220.1 |
| PFS months‡  (Range) | 0-153.0 | 0-170.4 | 2.0-171.0 | 2.2-101.9 | 4-231.4 | 1.0-220.1 |

* Patients without clinical information, follow-up data or *RRM2* expression level were excluded from this study.

# NKI data set used 25,000-gene array that comes from Agelent Technologies, which used same probers with Affymetrix HG-U133 array.

†OS: Overall survival

‡ PFS: Progression-free survival

**Supplementary Table 2. Demographic distribution of RRM2 in ZJU set**

|  | **RRM2** | | | **RRM2B** | | |
| --- | --- | --- | --- | --- | --- | --- |
|  | **High(%^*^)** | **Low** | ***p* value**† | **High(%*)** | **Low** | ***p* value**† |
| **Age** |  |  |  |  |  |  |
| <50 | 44(54.3) | 37 |  | 38(54.3) | 32 |  |
| ≥50 | 43(45.7) | 51 | 0.298 | 59(64.1) | 33 | 0.205 |
| **Histological type** |  |  |  |  |  |  |
| DC | 71(52.6) | 64 |  | 76(63.9) | 43 |  |
| SC | 10(38.5) | 16 |  | 13(44.8) | 16 |  |
| Other | 6(46.2) | 7 | 0.402 | 8(61.5) | 5 | 0.171 |
| **pT stage**^‡^ |  |  |  |  |  |  |
| T0-T1 | 24(42.9) | 32 |  | 35(72.9) | 13 |  |
| T2-T4 | 58(52.7) | 52 | 0.229 | 57(54.8) | 47 | 0.034 |
| **pN stage**^‡^ |  |  |  |  |  |  |
| 0 | 45(46.9) | 51 |  | 47(55.3) | 38 |  |
| 1-2 | 35(55.6) | 28 |  | 40(65.6) | 21 |  |
| 3 | 7(43.8) | 9 | 0.497 | 10(62.5) | 6 | 0.446 |
| **Grade** |  |  |  |  |  |  |
| 1=Well | 11(55.0) | 9 |  | 12(60.0) | 8 |  |
| 2=Mod | 38(50.7) | 37 |  | 42(62.7) | 25 |  |
| 3=Poor | 34(45.9) | 40 | 0.724 | 38(57.6) | 28 | 0.834 |
| **ER** |  |  |  |  |  |  |
| Negative | 33(51.6) | 31 |  | 28(51.9) | 26 |  |
| Positive | 50(52.1) | 46 | 0.949 | 64(68.1) | 30 | 0.050 |
| **PR** |  |  |  |  |  |  |
| Negative | 39(52.7) | 35 |  | 39(57.4) | 29 |  |
| Positive | 46(52.9) | 41 | 0.983 | 53(63.1) | 31 | 0.471 |
| **HER2** |  |  |  |  |  |  |
| Negative | 56(49.1) | 58 |  | 63(58.3) | 45 |  |
| Positive | 17(65.4) | 9 | 0.131 | 14(63.6) | 8 | 0.645 |
| **MKI67** |  |  |  |  |  |  |
| Negative | 27(40.9) | 39 |  | 31(48.4) | 33 |  |
| Positive | 58(59.8) | 39 | 0.018 | 61(67.8) | 29 | 0.016 |
| **Molecular subtype** |  |  |  |  |  |  |
| Luminal A | 23(46.0) | 27 |  | 27(52.9) | 24 |  |
| Luminal B/HER2 - | 19(55.9) | 15 |  | 24(72.7) | 9 |  |
| Luminal B/HER2 + | 7(70.0) | 3 |  | 5(55.6) | 4 |  |
| Basal-like TNBC | 17(65.4) | 9 |  | 15(55.6) | 12 |  |
| HER2-positive | 8(66.7) | 4 | 0.586 | 7(77.8) | 2 | 0.091 |
| **CD44/CD24 status** |  |  |  |  |  |  |
| CD44+/CD24- | 52(61.2) | 33 |  | 48(57.8) | 35 |  |
| Others | 33(45.2) | 40 | 0.001 | 40(63.5) | 23 | 0.750 |

Note: There are 1, 9, 15 14, 35m12 and 1 missing cases in Grade, pT stage, ER, PR, HER2,MKI67 and CD44/CD24 status. There are 132 cases could be classified on molecular subtype.

* % represent positive rate of RRM2-High or RRM2B_High it equal to N _High_/(N _High_+N_Low_)×100%.

‡ pT and pN stage are pathological stage. In this study, no patient has > or = 4 lympho nodes involvement.

† *p* values was based on Pearson Chi square test.
